# Family association study between INSR gene polymorphisms and PCOS in Han Chinese

**DOI:** 10.1186/1477-7827-9-76

**Published:** 2011-06-06

**Authors:** Xinghua Xu, Han Zhao, Yuhua Shi, Li You, Yuehong Bian, Yueran Zhao, Zi-Jiang Chen

**Affiliations:** 1 National Research Center for Assisted Reproductive Technology and Reproductive Genetics, China, The Key Laboratory for Reproductive Endocrinology of Ministry of Education Center for Reproductive Medicine, Provincial Hospital Affiliated to Shandong University, 324 Jingwu Road, Jinan, 250021, China

## Abstract

**Background:**

Polycystic ovary syndrome (PCOS) is a complex disease having both genetic and environmental components. Candidate genes with insulin metabolism have been hypothesized to be involved in the etiology of this syndrome. In the present study, we investigated the genetic association between polymorphisms in the insulin receptor (INSR) gene and PCOS.

**Methods:**

A total of 260 family trios were recruited and performed a family-based analysis to assess linkage and association between four single nucleotide polymorphisms (SNPs) (rs1799817, rs2059807, rs8108622 and rs10500204) of INSR gene and PCOS.

**Results:**

Using the transmission disequilibrium test (TDT), we failed to find that rs1799817 (p = 0.486), rs2059807 (p = 0.195), rs8108622 (p = 0.866) and rs10500204 (p = 1.0) were significantly overtransmitted to PCOS offspring from their parents.

**Conclusion:**

No significant evidence of association or linkage was found in the four tested markers, indicating that our family samples did not support susceptibility of the INSR gene to PCOS.

## Background

Polycystic ovary syndrome (PCOS), known as the most frequently encountered endocrinopathy in women, is a syndrome of chronic anovulatory, androgen excess and polycystic ovaries. It affects about 5-8% of child-bearing women[1]. This disorder is also associated with an increased risk of hyperinsulinemia, insulin resistance, type 2 diabetes mellitus, dyslipidemia, and cardiovascular diseases.

Previous studies have observed that PCOS is a polygene inheritance disease and has strong familial clustering, suggesting that PCOS may be a genetic disease [2-4]. Characteristics of PCOS such as elevated testosterone levels and polycystic ovaries have been regarded as autosomal dominant heredity [5-7]. Over the past decades, a number of candidate genes involved in insulin signaling pathway, steroid hormone synthesis, gonadotropin secretion and chronic inflammation have been performed to identify the susceptibility genes for PCOS [8,9]. However, the mode of inheritance for PCOS and the molecular mechanisms underlying PCOS have not been clarified. Several factors would complicate the molecular genetics of PCOS, such as population stratification, environmental factors and genetic heterogeneity. To avoid those impacting factors, it is crucial to study family-based analysis using the transmission disequilibrium test (TDT) to explore the putative contribution of candidate genes.

Genes involved in insulin action have been considered good candidates for PCOS [10,11]. Insulin resistance is present in the majority of PCOS cases. Women with PCOS have increased risk of type 2 diabetes [12], thus, INSR has been suggested as a good candidate gene of PCOS for the clinical features of PCOS. The INSR gene is located on chromosome 19 and composed of 22 exons [13]. It plays an important role in insulin metabolism. The tyrosine kinase domain mutations of the insulin receptor have been shown to cause severe hyperinsulinemia and insulin resistance [14-16]. In previous studies, the polymorphism rs1799817 of INSR gene has been proved to be associated with PCOS in Han Chinese and Caucasian [17,18]. Moreover, the SNPs rs2059807, rs8108622 and rs10500204, which arose from our recent work of large scale PCOS genome-wide association analyses, were also suspected to be associated with PCOS [18].

To further investigate the relationship between rs1799817, rs2059807, rs8108622 and rs10500204 in the INSR gene and the pathogenesis of PCOS, a family-based analysis was performed in 260 PCOS family trios of the present study. TDT was applied to assess linkage and association between PCOS and the candidate gene that may provide a better understanding of the contribution of INSR gene variation in the development of PCOS.

## Methods

### Subjects

Women with PCOS were recruited from the Center for Reproductive Medicine, Provincial Hospital Affiliated to Shandong University during the period from July 2007 to February 2010. PCOS was diagnosed according to the 2003 Rotterdam criteria [19], i.e., at least two of the following three features: oligomenorrhea or amenorrhea, clinical or biochemical hyperandrogenism and polycystic ovaries on ultrasound. Other related diseases, such as adrenal congenital hyperplasia, Cushing syndrome, and androgen-secreting tumors were excluded.

A total of 260 PCOS Han Chinese family trios [one affected daughter (proband) and both parents, 780 members] were recruited for this study. Informed consent for molecular studies obtained from all subjects, the study was approved by the Ethics Committees of Shandong University.

The mean age of 260 affected women was 27.42 years (27.42 ± 4.02 in mean ± standard deviation (SD)) and the mean body mass index (BMI) was 25.13 kg/m^2 ^(25.13 ± 4.29 in mean ± SD). The mean levels of total testosterone were 61.25 ng/dl (61.25 ± 33.86 in mean ± SD). Among all the probands, the mean levels of fasting glucose and fasting insulin were 5.47 mmol/L (5.47 ± 1.31 in mean ± SD) and 11.09 mIU/L (11.09 ± 6.95 in mean ± SD), respectively (Table 1).

**Table 1:** Clinical and metabolic characteristics of PCOS patients

	Mean	SD
Age (year)	27.42	4.02
BMI (kg/m2)	25.13	4.29
TT (ng/dl)	61.25	33.86
FG (mmol/L)	5.47	1.31
FINS (mIU/L)	11.09	6.95

SD, standard deviation; TT, Total testosterone; FG, Fasting glucose; FINS, Fasting insulin

### Genotyping

A 5 ml whole-blood sample was collected for each subject. Genomic DNA was extracted using a QIAamp DNA mini kit (QIAGEN, Hilden,, Germany) according to the manufacturer's protocol. Four valid SNPs (rs1799817, rs2059807, rs8108622 and rs10500204) in the INSR gene were amplified using polymerase chain reaction (PCR) with three pairs of INSR-specific primers (rs8108622 and rs10500204 share a pair of primers). Positional information of the four SNPs was shown in Table 1. The following PCR primers were used for rs1799817: 5' -GGTCAACGAGTCAGCCAGTCT-3' (sense), and reverse 5'- TCCAGAAAGTGATGAGACAGTGAT -3' (antisense); for rs2059807: 5' - GACCCAGTATGCCATCTTTGTG -3' (sense), and reverse 5'- TGCTTGAGCCCAGGAGTTTG -3' (antisense); and for rs8108622 and rs10500204: 5' - GTCCCAGATACCAAGGATGTGC -3' (sense), and reverse 5'- GAGAATTAGCCAAGCGAGAGTGT -3' (antisense). The reaction was carried out under the following conditions: initial denaturation at 95°C for 5 min followed by 35 cycles of denaturation at 94°C for 30 s, annealing for 30 s at 60°C, extension at 72°C for 1 min, and finally 72°C for 7 min. The PCR products were first analyzed by 1% agarose gel electrophoresis (AGE) and then sequenced on an automated sequencer (ABI PRISM 310; Applied Biosystems, Foster City, CA).

### Statistical analysis

Descriptive statistics for individual SNPs, including minor allele frequency (MAF), Hardy-Weinberg equilibrium as well as linkage disequilibrium (LD) statistics between SNPs (D', r^2^) were obtained from Haploview 4.2 [20]. Then association between the four SNPs and PCOS was tested by the TDT analysis which was performed using Haploview 4.2 [20]. Statistical significance was considered at the two-tailed P level of 0.05. The TDT is a valid Chi-square test statistic for the linkage hypothesis, regardless of population history. In the TDT test, by collecting unrelated PCOS family trios, we analyze the difference between the probability of parents-to-offspring transmission and the hypothesis of no association (probability of transmission 50%). If the discrepancy exists, the reason would be the association between INSR polymorphisms and PCOS.

The haplotype structure for SNPs in rs8108622 and rs10500204 (only these two SNPs were in strong linkage disequilibrium) was constructed and analyzed with Haploview 4.2 [20].

## Results

### MAFs and TDT analysis

A total of 260 families were used for the analysis. The MAFs of these SNPs were 0.377(rs1799817), 0.313 (rs2059807), 0.153 (rs8108622) and 0.153 (rs10500204), respectively, and the four SNPs were in Hardy-Weinberg equilibrium (p > 0.05) (Table 2).

**Table 2:** Positional Information and summary statistics for SNPs in the INSR gene

Variant	Location	**Position**^**a**^	**Alleles**^**b**^	HWE p	MAF
rs1799817	Exon 17	197125297	C/**T**	0.957	0.377
rs2059807	Intron 8	197166109	T/**C**	0.193	0.313
rs8108622	Intron 3	197182753	T/**A**	0.480	0.153
rs10500204	Intron 3	197182963	A/**C**	0.480	0.153

^a ^Contig accession number is NT_011255.14; ^b ^Minor allele in bold

MAF, Minor allele frequency

TDT analysis was only performed with at least one parent being heterozygous, so in the all 260 nuclear families, 249 trios of rs1799817, 238 trios of rs2059807, 140 trios of rs8108622, 140 trios of rs10500204 were entered the TDT analysis. Results from TDT were shown in Table 3. No significant transmission disequilibrium between the alleles of the four SNPs and PCOS was found (rs1799817: χ^2 ^= 0.486, p = 0.486; rs2059807: χ^2 ^= 1.681, p = 0.195; rs8108622: χ^2 ^= 0.029, p = 0.866; rs10500204: χ^2 ^= 0, p = 1.0; see Table 3). The results indicated that the four SNPs may not participate in the pathogenesy of PCOS.

**Table 3:** TDT results for the four SNPs in INSR gene

Marker ID	Overtransmitted allele	T	Not-T	Total TDT	Transmission frequency	**TDT χ**^**2**^	P-value
rs1799817	C	130	119	249	0.522	0.486	0.486
rs2059807	C	129	109	238	0.542	1.681	0.195
rs8108622	T	71	69	140	0.507	0.029	0.866
rs10500204	-	70	70	140	0.500	0	1.0

T, number of transmissions in TDT analysis

### Haplotype analysis

Linkage disequilibrium was also analyzed in the subjects. Only rs8108622 and rs10500204 were in strong LD (r^2^= 0.93, D' = 0.965), so haplotype analysis was pursued. However, the haplotypes did not correlate well with association with PCOS, as determined by TDT (Table 4).

**Table 4:** TDT analysis for the SNP haplotypes in INSR gene

**SNP haloptype allele**^**a**^	Allele frequency	T	Not-T	Total TDT	Transmission frequency	**TDT χ**^**2**^	P-value
A.TA	0.841	72	69	141	0.511	0.064	0.801
B.AC	0.150	68	67	135	0.504	0.007	0.931

^a ^Order of SNPs in haplotype: rs8108622, rs10500204.

T, number of transmissions in TDT analysis

## Discussion

As a complex multigenic and heteroplasmy disease, various susceptibility genes of PCOS interact with each other and the environmental factors also influence the occurrence and development of the syndrome [21]. Concerning molecular genetic studies, PCOS is one of the most extensively studied endocrinopathys in women, and attention has been given to insulin resistance, with special focus on the INSR gene, with uncertain results. Therefore, in this study, we used family-based analysis in order to investigate the relationship between the INSR gene SNPs and the genetic component of PCOS.

Our data showed that the MAFs of these SNPs were 0.377(rs1799817), 0.313 (rs2059807), 0.153 (rs8108622) and 0.153 (rs10500204), respectively, consistent with the common population (NCBI) and our recent research data. The MAFs in NCBI were 0.427 (rs1799817), 0.28 (rs2059807), 0.126 (rs8108622) and 0.129 (rs10500204), respectively. And the MAFs of our recent research cases were 0.408(rs1799817), 0.342 (rs2059807), 0.178 (rs8108622) and 0.179 (rs10500204), respectively [17,18]. Thus there was no selection bias of our subjects.

The SNP in exon 17 of the INSR gene, in the tyrosine kinase domain of the insulin receptor have been paid more attention. Siegel et al [22]found an association between rs1799817 and PCOS. They argued for the first time that the INSR gene itself took part in the development of PCOS. Similarly, we also found the polymorphism itself may predispose to the development of PCOS [17]. In India, the researchers found an association of rs1799817 with indices of insulin resistance and hyperandrogenemia in the same subgroup [21]. Not corroborating previous studies, we failed to find that rs1799817, rs2059807, rs8108622 and rs10500204 were significantly over-transmitted to PCOS offspring from their parents by using a genetic approach of association analysis. Moreover, the analysis of the haplotypes did not show evidence of association between these polymorphisms and PCOS. The results of the analysis of individual alleles, as well as the analysis of haplotypes, appeared to exclude a direct role of rs1799817 in exon 17, rs2059807 in intron 8, rs8108622 and rs10500204 in intron 3 at the INSR gene in the pathogenesis of PCOS in our sample. As well as a case-control association study carried out in Korean population, they also failed to provide evidence for the association between INSR gene and PCOS [12].

The discrepancy between these studies may be caused by population stratification. Considering using the classic case-control design would have the limitation that associations between allelic variants in candidate genes and disease might have arisen on account of population stratification by ethnicity or environmental factors [23], a family-based association study (case-parent trios) was used in the present research rather than unrelated cases and controls (case-control study). Using the TDT analysis, a particularly appropriate test for a possible role of a candidate gene [10] in a family-based association study, we can get rid of the influence of population stratification. The result indicated that these polymorphisms at the INSR gene were unlikely to be relevant in the development of PCOS in our sample.

In addition, as environmental factors are also involved in PCOS [24,25], the interaction with gene may be a key point of the pathogenesis in PCOS, not only the gene itself. When we used the family trios analyzing the subjects' genotypes, the interaction would be exorcized. So that may be one reason for we did not find any association between the four SNPs and PCOS.

Our results also showed that the MAFs of both of rs8108622 and rs10500204 were only 15.3%, therefore, the number of families that could be analyzed using TDT was small (only 140). Thus, we speculated that negative result of our association study of INSR gene with PCOS might due to the low frequency of the two SNPs in Chinese Han population. And a larger scale family-based research should be carried out to identify the role of the SNPs in PCOS. Furthermore, because of the highly polymorphic of INSR molecule, further investigations are needed to address the contribution of other SNPs and several polymorphisms in the form of haplotypes [26]. There is the possibility that molecular defects in other genes that regulate the expression of the INSR gene could be involved in the pathogenesis of PCOS.

Our study covered a relatively large series of PCOS trios and represents the first family association study providing data about the INSR gene and PCOS in a Chinese Han population. Finally, we have to mention that PCOS is a multi-factorial disease, even though our data showed no evidence that INSR gene polymorphism increases the risk of developing the syndrome, further investigations with an increased number of subjects and more SNPs are necessary.

## Conclusions

In the present study the relationship between the INSR gene SNPs and the pathogenesis of PCOS was investigated in 260 PCOS family trios. Using the transmission disequilibrium test, we failed to find that rs1799817, rs2059807, rs8108622 and rs10500204 were significantly over-transmitted to PCOS offspring from their parents. Our family based research did not support susceptibility of the INSR gene to PCOS and further investigations with an increased number of subjects and more SNPs are necessary.

## Competing interests

The authors declare that they have no competing interests.

## Authors' contributions

XX participated in the design of the study, collected the materials, carried out all experiments and drafted the manuscript. HZ, YS, YZ, and Z-JC participated in the design of the study and helped to draft the manuscript. LY and YB helped to carry out PCR and collected the materials. All authors read and approved the final manuscript.
